# Knowledge and practice of breast self-examination among breast cancer patients in Damascus, Syria

**DOI:** 10.1186/s12905-024-02912-8

**Published:** 2024-01-28

**Authors:** Mohammed Alshafie, Jameel Soqia, Dima Alhomsi, Mhd Basheer Alameer, Laila Yakoub - Agha, Maher Saifo

**Affiliations:** Faculty of Medicine, Damascus University, Damascus, Syrian Arab Republic

**Keywords:** Breast cancer, Breast self-examination, Clinical breast examination, Mammography

## Abstract

**Background:**

Breast cancer (BC) represents an important cause of cancer death, its incidence rate has been rising gradually in the Arab world, and in Syria, BC is the most common cancer and the leading cause of cancer death; its prognosis gets better as we detect it early in its first stages. So, it is very important to implement one or more early detection methods such as Breast Self-Examination (BSE), Clinical Breast Examination (CBE), and mammography. BSE represents an effective method to find out changes in breast structure when they happen. This study investigates the knowledge of BSE and its practice in BC patients.

**Methods:**

A quantitative cross-sectional study in Al-Bairouni hospital in Damascus-Syria was carried out using face-to-face interviews based on a structured questionnaire, which consisted of 4 sections. The data were subjected to statistical analysis using various analytical tests, including the independent t-test, one-way analysis of variance (ANOVA), and Chi-square test.

**Results:**

Five hundred patients were interviewed. Only 27.4% of patients had a good knowledge of BSE, 17.4% had average knowledge, and 55.2% had low knowledge of BSE. The factors that have an impact on the knowledge of BSE were: family breast cancer history (first and second-degree relatives), education, and the region of living (between governorates). The effect of knowledge of BSE on its practice was positive. However, only 24.8% of patients have been practicing BSE; the reasons for not practicing BSE were: no one has told the patient about it (64.8% of cases), and the patient does not have any symptoms relating to the breast (21.4%).Breast cancer was identified through breast self-examination (BSE) in 15.6% of cases.

**Conclusion:**

There is a low degree of knowledge and little practice of BSE among Syrian breast cancer patients. Family breast cancer history, governate, occupation, and level of education had a statistically significant effect on knowledge scores of BSE, unlike age and social status. So, some steps should be taken to increase awareness about BSE among Syrian females.

## Background

Cancer is a leading cause of death worldwide, responsible for nearly 10 million deaths in 2020 [1]. Breast cancer (BC) is the most common cancer globally, with 2.26 million cases in 2020 and representing the fifth leading cause of cancer deaths, accounting for 685,000 deaths [1]. Although the incidence rate of BC is lower in developing countries compared to the Western world [2]. it has been rising gradually in the Arab world since 1990, and it is expected to increase further over the next 10 years without intervention [3], In the Arab world, 11 out of 100,000 deaths among women are BC-related [3]. In Syria, BC is the most common cancer, accounting for nearly 21% of all new cancer cases and is the leading cause of cancer death as of 2020 [4].

Early detection and effective treatment are critical for the prognosis of BC, with stage 0 and stage I patients having a 100% 5-year survival rate. The 5-year survival rate for stage II and stage III breast cancer patients is 93% and 72%, respectively. However, when the tumor metastasizes, its prognosis worsens significantly, with only 22% of stage IV BC patients surviving for 5 years [5], Psychological symptoms can also burden BC patients [6], underscoring the importance of early screening and diagnosis to decrease morbidity and mortality [7]. Breast Self-Examination (BSE), Clinical Breast Examination (CBE), and mammography are among the many screening methods for BC [8]. However, several studies have found a lack of screening among Arab women [9, 10], and BC is usually diagnosed at advanced stages [11, 12]. The Centers for Disease Control and Prevention (CDC) recommends mammography every two years for women aged 40 to 74 to screen for BC [13]. However, due to the Syrian crisis, many women cannot undergo CBE or mammography, even though mammography is the best way to detect breast cancer early [13], especially those who live in developing cities or rural areas of the country [14], which makes BSE; a cost-effective and easily performed method, it involves self-administration by women on a monthly basis, usually one week after menstruation, without the need for medical expertise or a visit to a healthcare facility [15]. However, it has not been proven as the best method for early detection [13]. A similar study in Turkey found low levels of knowledge and practice among women pre-training [16], while a systemic review discussing BSE in African women suggested various levels of knowledge, attitude, and practice, with nearly half the studies suggesting good levels of them [17]. Despite the potential benefits of BSE, there are no articles discussing the assessment of BSE’s knowledge and practice among Syrian breast cancer patients. Therefore, this study aimed to assess BSE knowledge (including knowledge of the examination itself and time intervals between each examination) in relation to demographic characteristics, previous practice of BSE among BC patients, and the reason for not practicing it if not practiced. The study was conducted in Al-Bairouni university hospital in Damascus – Syria, which is the only center specializing in treating cancer patients in the country [14].

## Methods

### Study design and sampling

This study is a quantitative cross-sectional study that was conducted among Syrian women diagnosed with breast cancer who visited Al-Bairouni Hospital in Damascus, Syria. The sample size (n) of 500 was determined using Cochran’s Sample Size Formula with a 95% confidence level (Z = 1.96), a margin of error of 5%, an estimated proportion of the population with the attribute in question of 50% (or 0.5), and q of 1-p. Face-to-face interviews were conducted from 15/11/2021 to 18/12/2021, and a convenience sampling method was used to achieve the sample size. Ethical approval to conduct this study was obtained from the administration of Damascus University.

### Data collection

The research team collected data via face-to-face interviews based on a structured questionnaire. We administered the questionnaire to patients in drug administrating rooms while maintaining the secrecy of information after obtaining verbal consent from participants. The average time for each interview was 7–10 min. All women diagnosed with breast cancer who attended the hospital were included, while the inclusion criteria encompassed women who declined participation, responses with incomplete data, and poor health status observed in patients aged 75 years and above.

### Questionnaire

Our questionnaire was adapted from similar previous studies [18], and [19] as a point of reference for validation.

### The questionnaire consisted of 4 sections

Section 1 included Sociodemographic characteristics (Age, Marital status, Occupation, Education, governorate of origin, and Family history of breast cancer).

Section 2 evaluated participants’ knowledge about BSE by asking several questions about the method of BSE, how often and when it should be performed, and the recommended age to start performing it.

Section 3 evaluated their practice of BSE; whether they performed BSE, how often they performed it, or the reason for not performing it.

Section 4 asked about other undergoing ways of detecting breast cancer ( CBE and Mammography).

Questions varied between Yes/No questions and multiple-choice questions.

The questionnaire was pre-validated and used in similar previous studies, including its Arabic version [18, 19]. We adopted a test to assess its reliability in evaluating participants’ knowledge and practice of BSE. Points were given as one point for the correct answer and zero for the incorrect one. Cronbach’s Alpha coefficient was measured and showed an acceptable value of 0.93 for the knowledge section and 0.77 for the practice section.

### Ethics approval

The study was in compliance with the Declaration of Helsinki for research involving human subjects. The Ethical Committee approved this study in the Faculty of Medicine at Damascus University, Syria (9197, 7-11-2021). All our methods were carried out following relevant guidelines and regulations. Informed consent to participate and verbal consent was taken with patients’ signatures, this was approved by the ethical committee of Damascus University (ID: 9197). We explained the purpose of the study to each participant and the way to answer the questionnaire and it was all voluntary.

### Statistical analysis

We used the Kobo toolbox app to gather data and then entered them into an Excel sheet imported to SPSS version 25 for data analysis. Data were summarized using descriptive statistics including absolute and relative frequencies, median, and percentage. An Independent t-test was used to measure the effect of family breast cancer history on the knowledge of BSE. One-way ANOVA was used to compare the educational level, the governorate of origin, occupation, age, and social status on the knowledge of BSE. The Chi-square test was used to measure the effect of family breast cancer history, age, the governorate of origin, and educational level on the practice of BSE. The effect of practicing BSE on the knowledge of it was also studied using an independent t-test. A P value of < 0.05 was considered statistically significant.

## Results

A total of 501 patients have been interviewed, and one of them was excluded because of missing data. 310 (62%) of patients were older than 45 years, whereas 188 (37.6%) were between 25 and 45 years old, and only 2 (0.4%) were less than 25 years old. 14.8% of patients had a first-degree relative diagnosed with breast cancer, while 23.6% had a second-degree relative diagnosed with breast cancer. Other sample details and Characteristics are shown in Table 1.

**Table 1:** Sample characteristics

Variables		Frequency	Percent
Age	Less than 25 years	2	0.4
	Between 25–45 years	188	37.6
Social status	Older than 45 years	310	62.0
	Single	52	10.4
	Married	402	80.4
	Divorced	12	2.4
	Widow	34	6.8
Education level	Uneducated	83	16.6
	School (primary, secondary) only	305	61.0
	College student	20	4.0
	Graduated	89	17.8
	Master’s degree	3	0.6
Occupation	Employee	118	23.6
	Housewife	381	76.2
	Student	1	0.2
Governate	Damascus (capital)	100	20.0
	Rif Dimashq	83	16.6
	Daraa	39	7.8
	AL-Qunaitera	16	3.2
	Al-swidaa	27	5.4
	Homs	46	9.2
	Hama	51	10.2
	Aleppo	34	6.8
	Idlep	2	0.4
	Lattakia	3	0.6
	Tartus	11	2.2
	Der ALzour	40	8.0
	Al-Hasaka	34	6.8
	AL-Riqa	14	2.8
Family breast cancer history	Had a first-degree relative	74	14.8
	Had a second-degree relative	118	23.6

**Fig. 1:**
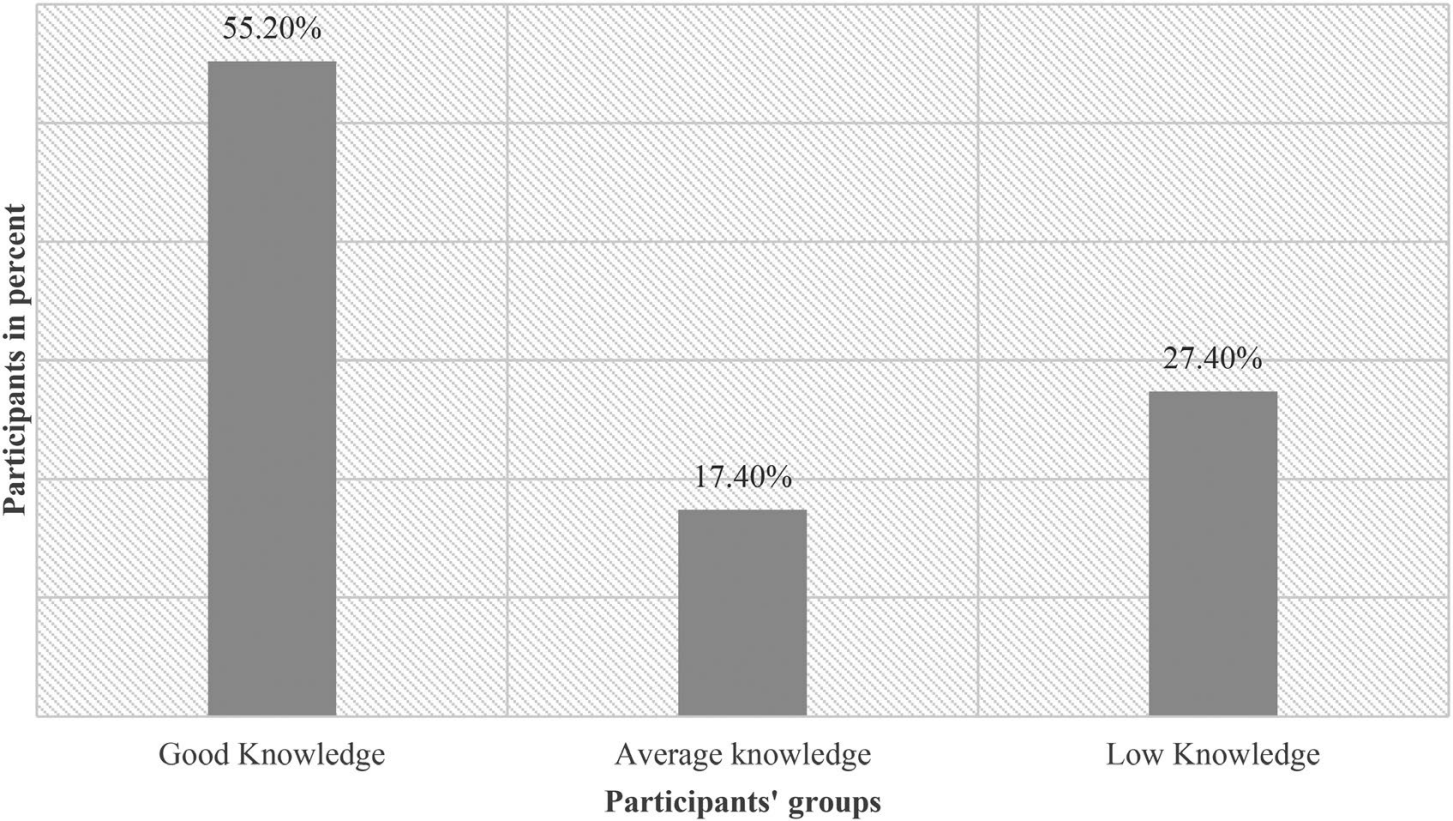
Knowledge of BSE scores distribution

Mean test results for the knowledge of BSE were 3.37 out of 10; only 27.4% of patients had a good knowledge of BSE (their test results were from 7 to 10), while 17.4% had average knowledge of BSE (test results were from 4 to 6). However, 55.2% of patients had low knowledge of BSE (test results were below 4) (Fig. 1). Each question’s results and other details are shown in Table 2.

**Table 2:** BSE questions test results

BSE test	Answers	
	Correct	Not correct
Have you heard of BSE	Yes = 54.2%	45.8%
Do you know that BSE is a useful tool for early detection of breast cancer	Yes = 49.4%	50.6%
At what age should BSE be started	From 20 years = 11%	89%
How often should BSE be done	Monthly = 14.6%	85.4%
What is the best time to do BSE	A week after menstruation = 29.4%	70.6%
BSE should be done by	The woman herself = 43.2%	56.8%
BSE is done by inspecting the breast in the mirror	Yes = 32%	68%
BSE is done by feeling the breast and the armpit with the hand	Yes = 41.8%	58.2%
BSE is done by doing Ultrasound of the breast	No = 30.8%	69.2%
BSE is done by mammography	No = 30.2%	69.8%

This study found that participants, who had a Family Breast Cancer History (First-degree relatives), had statistically significantly higher Knowledge scores of BSE (M = 4.24 out of 10) compared to participants who had not (M = 3.21), t(498) = 2.31, *p* < 0.001. Also, participants, who had a family breast cancer history (second-degree relatives), had statistically significantly higher Knowledge scores of BSE (M = 3.97 out of 10) compared to participants who had not (M = 3.18), t(498) = 2.13, p-value = 0.033.

**Fig. 2:**
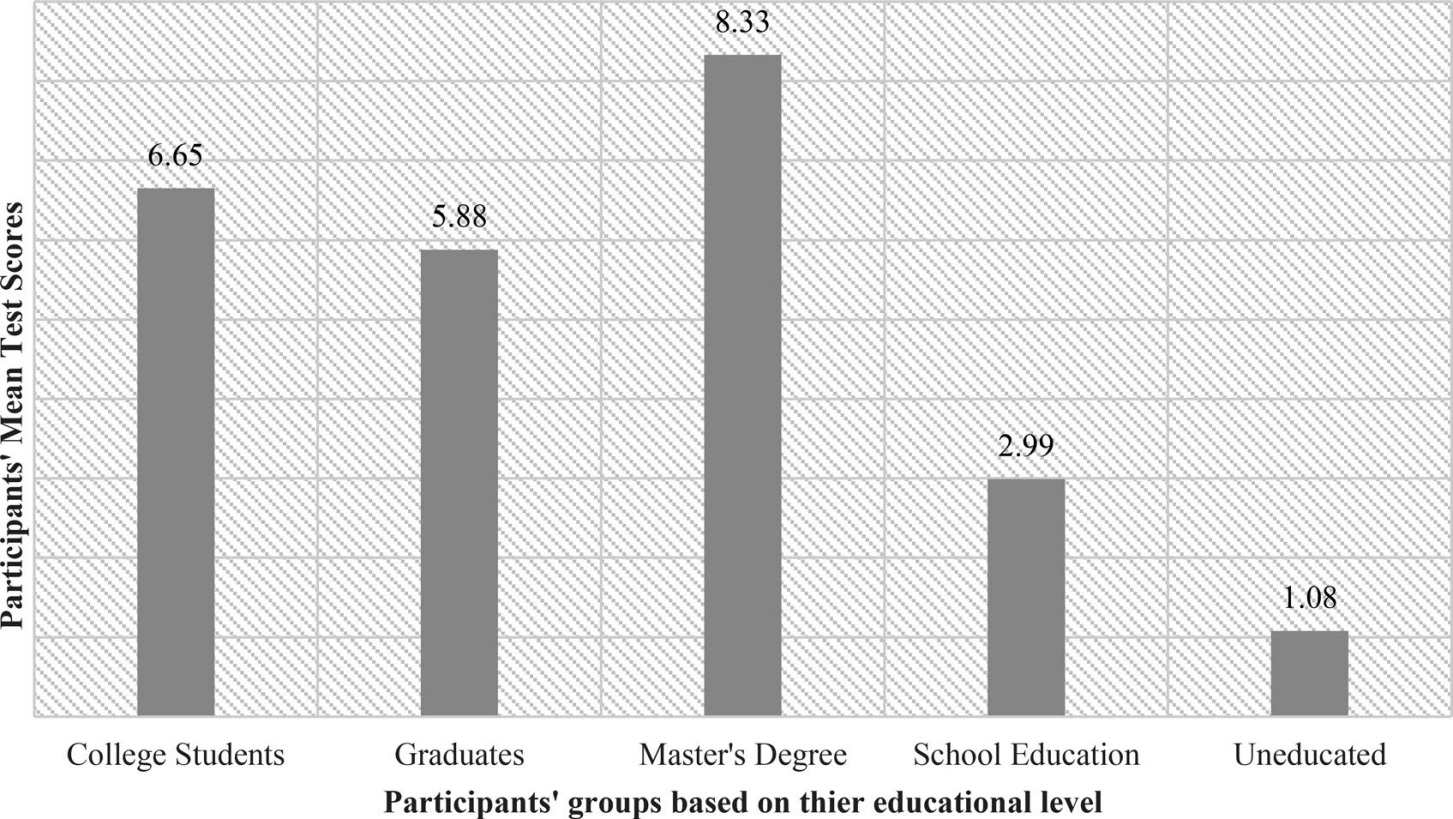
Education impact on knowledge of BSE

A one-way ANOVA was performed to compare the effect of educational level on the Knowledge of BSE, there was a statistically significant difference between groups as determined by one-way ANOVA (F (4,498) = 32.91, *p* < 0.001). A Tukey post hoc test revealed that the Knowledge of BSE scores was statistically significantly lower in the school education group (M = 2.99, *p* < 0.001) and illiterate patients (M = 1.08, *p* < 0.001) compared to the College students, graduates, and master’s students’ groups (M = 6.65, M = 5.88, and M = 8.33, respectively) (Fig. 2).

**Fig. 3:**
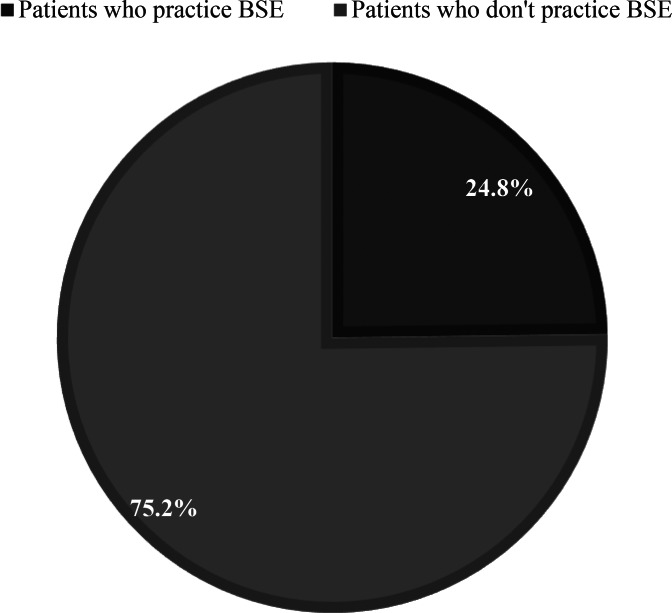
Practicing the BSE rates

Additionally, a one-way ANOVA was performed to compare the effect of Governate (the place of living) on the Knowledge of BSE, there was a statistically significant difference between groups as determined by one-way ANOVA (F (13,498) = 5.65, *p* < 0.001). A Tukey post hoc test revealed that the Knowledge of BSE scores was statistically significantly lower in the Aleppo group (M = 1.88, *p* < 0.001), the Der alzour group (M = 1.55, *p* < 0.001), and Al-Hasaka Group (M = 1.82, *p* < 0.001) compared to Damascus (the capital) group (M = 4.31). Also, Occupation had a significant effect on the Knowledge of BSE, F (2,498) = 38.19, *p* < 0.001. Employed patients had better Knowledge of BSE scores than Housewives; test results means were 5.69 and 2.65, respectively. A one-way ANOVA was performed to compare the effect of Age, and social status on the Knowledge of BSE. There was no statistically significant difference between different age groups or between different social status groups with a p-value of 0.546 and 0.059, respectively. Only 24.8% of patients have been practicing BSE (Fig. 3), 5% of patients were practicing BSE once a month, 2% were practicing it once every 3 months, 7% were after each shower, and 9.8% were practicing BSE irregularly. 1.4% of patients started doing BSE at an age of fewer than 25 years, 3% at an age between 25 and 30 years, 7.2% at an age between 30 and 35 years, and 13.2% started after the age of 35.

**Fig. 4:**
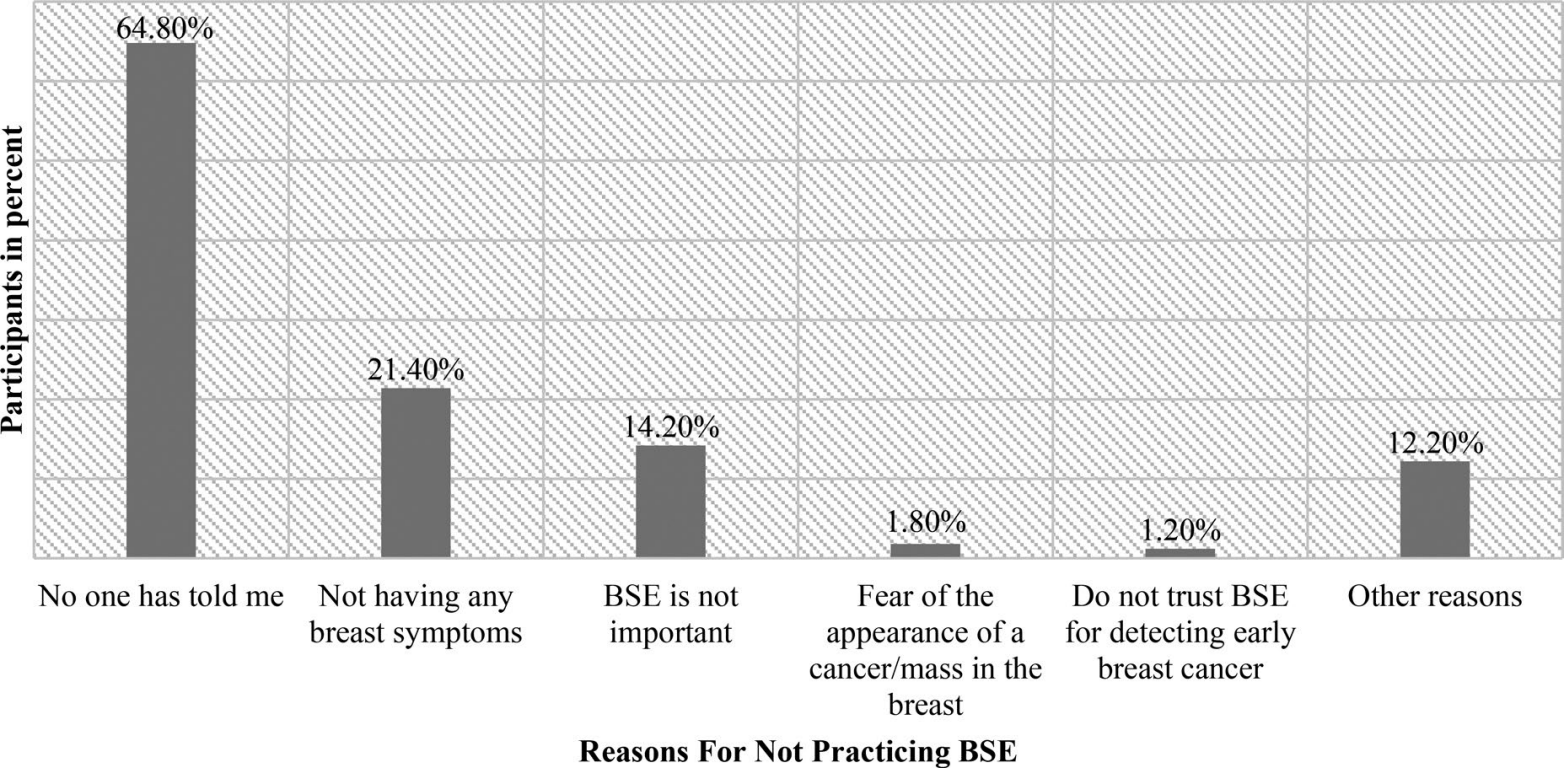
Reasons for not practicing BSE

For the reasons why you didn’t practice BSE, 64.8% of patients stated that no one has ever told them about BSE and they had never heard about it, 21.4% of patients attributed the cause to not having any breast symptoms or problems, 14.2% thought that BSE is not important, 1.8% attributed the cause to their fear from the appearance of cancer or a mass in the breast, 1.2% did not trust BSE for detecting early breast cancer, and 12.2% attributed the cause for other reasons (Fig. 4).

There is a significant relationship between family breast cancer history (First-degree relatives) and the Practice of BSE, X2(1, *N* = 500) = 7.91, *p* = 0.005. Patients who had a first-degree family breast cancer history are more likely to practice BSE than patients who didn’t have a first-degree family breast cancer history (37.8–22.5%). Also, there is a significant relationship between family breast cancer history (Second-degree relatives) and the Practice of BSE, X2(1, *N* = 500) = 8.19, *p* = 0.004. Patients who had a second-degree family breast cancer history are more likely to practice BSE than patients who didn’t have a second-degree family breast cancer history (34.7–21.7%). However, there is no relationship between the age of the patients and their practice of the BSE, while there is a significant relationship between the region of living and the Practice of BSE, X2(13, *N* = 500) = 43.17, p-value < 0.001. Patients who lived in the city and the capital of the country (Damascus) are more likely to practice the BSE than those who live in remote areas or regions.

However, practicing BSE had a significant effect on the Knowledge of BSE, t(498) = 17.24, p-value < 0.001. Patients who practice BSE had a greater knowledge of BSE than those who don’t practice BSE (7.15 to 2.12 mean BSE Knowledge test results). Also, there is a significant relationship between the education level and the practice of BSE, X2(4, *N* = 500) = 65.76, p-value < 0.001. Patients who went to college are more likely to practice BSE than those who didn’t or those who only went to school (46.1–21.4%).

Regarding the detection of breast cancer in these individuals, 78 patients identified it through breast self-examination (BSE), 20 patients through clinical breast examination (CBE), 13 patients through regular mammography screenings, and finally, 389 patients noticed breast changes and sought medical attention.

## Discussion

In relation to the ongoing Syrian crisis and the escalating costs of breast cancer detection equipment such as ultrasound and mammography, breast self-examination (BSE) has become crucial for Syrian women, despite its recognized limitations in early detection efficacy [8, 20].

Our study focused on Syrian breast cancer patients and revealed a concerning lack of understanding regarding the fundamentals of BSE, with an average score of 3.37 out of 10 on the knowledge test. Nevertheless, approximately half of the patients were aware of BSE, a significantly higher percentage compared to a similar study conducted among breast cancer patients in Egypt [21] where only 10.4% were aware. Similar studies among healthy women in Saudi Arabia [22] (30%), and Vietnam (22.7%) [23], reported lower awareness rates, while a study in Palestine [24] reported a higher rate of 76.7%. However, among those familiar with BSE, there was a notable lack of information regarding its timing, recurrence, and technique, which aligns with findings from the Saudi Arabian study [22]. It has been suggested that all women should practice BSE starting from the age of 20 [25]. However, our study indicated a prevailing misconception in the Syrian community that younger women cannot be diagnosed with cancer. The majority of participants agreed that women should start BSE after the age of 30, and among those practicing BSE, 55.3% started it after the age of 35. Furthermore, most women who claimed to practice BSE did so irregularly, rather than monthly as recommended. This pattern resembled findings from Vietnam [23] and Iran [26]. In contrast, other studies conducted among nurses or university students, who have better access to medical information, reported a higher percentage of women correctly identifying the ideal age and timing for BSE [27, 28]. Approximately one-third of Syrian breast cancer patients demonstrated awareness of breast changes during the menstrual cycle and agreed that BSE should be performed after the end of the period. Interestingly, there was a significant disparity between the number of women who had heard of BSE (54.2%) and those who knew how to perform it correctly (28%), highlighting a common confusion between periodic radiographic examinations and BSE. Similar findings were reported in a previous study [29] where only 0.7 of Hispanic women were proficient in BSE techniques. Additionally, when asked about the steps of BSE, women were more confident in breast palpation than in observing their breasts in front of a mirror. In contrast, 70% of nurses in an Emirate study were certain of all BSE steps [28], indicating that Syrian women have access to information about BSE but require more guidance.

Our study aimed to assess whether women could differentiate between BSE and periodic ultrasound and mammogram examinations. Only 29.8% of participants answered correctly, suggesting a common confusion between these radiographic examinations and BSE.

Women with a positive family history of breast cancer displayed better knowledge and practice of BSE compared to others, which can be attributed to their firsthand experience with symptoms, screening methods, and treatment protocols. Similar results were mentioned in Ethiopian [30] and Egyptian studies [21]. Additionally, uneducated women and housewives’ knowledge of BSE was significantly lower than the others, respectively. The latter can be due to the lower capability of searching for information resulting in insufficient knowledge of diseases, as suggested in a Vietnamese study [23], less social interaction, especially for housewives [21], regardless of their level of education, and Illiteracy which is represented in the inability to read; thus, written methods of spreading awareness are considered to be useless in that case. Our participants’ scores were significantly lower than those of university students in a previous study conducted at the Syrian Private University [10] and other studies among women with higher education [26, 31]. Interestingly, when analyzing the results, the correlation between having any education and BSE knowledge was found to be stronger than that between having a family history of cancer and BSE knowledge, which is consistent with the finding of an Indian study [32]. Our results showed that women in rural regions and especially remote regions had significantly lesser knowledge of BSE than women in urban regions, and were less likely to practice it which can be attributed to the fact that rural regions have less efficient educational systems and many early marriage cases, which indeed forces the female to drop out of school [33], and in comparison with the Ethiopian study [30] which was among university students, the previous result was still true despite the level of education, probably due to the lack of awareness campaigns in rural regions compared to the capital and nearby regions and more importantly, the lack of technology means which are considered a useful way of spreading awareness [34]. Surprisingly, age and social status did not significantly impact BSE knowledge or willingness to practice it, contrary to findings in other studies where older adults were more likely to perform BSE [35, 36].

Out of the 500 patients surveyed, only 24.8% claimed to have performed BSE before their breast cancer diagnosis. This figure is higher than the reported BSE rates in Saudi Arabian studies (19%) [22], and Egypt [21] (2.6%), but still significantly lower than the Palestinian study [24] where 40% of participants practiced BSE. When women who did not practice BSE were asked about the barriers preventing them, the most common answers included not being informed about BSE, the absence of breast symptoms or problems, and a perception that BSE was unimportant. In contrast, a study conducted in Turkey found that young age was a reason for not practicing BSE due to a perceived low risk of breast cancer [36], while the main barrier in the Palestinian study was a lack of knowledge and misconceptions about BSE [24].

As expected, there was a correlation between BSE knowledge level and its practice. Women with higher scores were more likely to perform BSE, regardless of the technique used. Conversely, women who already practiced BSE scored higher on the knowledge test, with an average score of 7 points. This finding aligns with a previous study in Ghana that discussed theimportance of knowledge in promoting BSE practice [37]. Surprisingly, our findings have demonstrated a significant association between breast self-examination (BSE) and the detection of breast cancer among Syrian breast cancer patients. Among the 500 patients included in the study, 78 patients were able to identify their cancer through BSE, whereas a smaller number of patients detected their cancer through periodic mammography or Clinical Breast Examination (CBE). This discrepancy may be attributed to the challenges associated with adhering to regular mammography or CBE, potentially due to the high cost involved, which diminishes the perceived benefits of these methods. Furthermore, this was corroborated by the responses obtained when patients were asked about the frequency of their CBE screenings; the majority reported irregular screenings, primarily prompted by the sudden observation of breast changes or during the performance of BSE.

## Limitations

The results of this study relied on the honesty of the participants’ responses and their ability to retrieve memories about their daily actions despite their psychological state, which may lead to information bias. Additionally, most of the respondents belonged to the older population; thus, this study did not give much information about the knowledge and the practice of BSE among younger breast cancer patients. Our sample size was representative of breast cancer patients visiting Al Birouni Hospital during the last year, but a larger sample and a longer data collection time can be used for better and more accurate results. Moreover, patients visiting Al Birouni Hospital do not represent the entire country’s breast cancer patient population, so more studies are for results more representative of the Syrian breast cancer patient population in the whole country.

## Recommendations

We propose several pragmatic measures to enhance awareness of this examination among patients and the wider population, such as: Introducing women to BSE on national TV stations, setting up educational seminars for patients in Al-Birouni hospital to introduce them to BSE benefits and how to perform it and asking them to teach their first relatives (daughter, sister, mother) about it, sharing articles about BSE by social media medical persona.

## Conclusion

Our Data showed a low degree of knowledge and little practice of BSE among breast cancer patients, the main reason for these results is no one has ever told them about BSE. Family breast cancer history (First and second-degree relatives), governate (the place of living), and level of education had a statistically significant effect on knowledge scores of BSE and practice BSE. Also, the occupation had a positive effect on the knowledge of BSE scores. On the other hand, there was no effect of age or social status on knowledge. There is a great need to disseminate correct information about breast self-examination in Syria.

## Data Availability

The datasets generated during and/or analyzed during the current study are available from the corresponding author on reasonable request.

## Abbreviations

BSE: Breast Self-Examination
CBE: Clinical Breast Examination
BC: Breast Cancer
